# Proteomics-based identification of TMED9 is linked to vascular invasion and poor prognoses in patients with hepatocellular carcinoma

**DOI:** 10.1186/s12929-021-00727-5

**Published:** 2021-04-22

**Authors:** Yi-Chieh Yang, Ming-Hsien Chien, Tsung-Ching Lai, Min-Che Tung, Yi-Hua Jan, Wei-Ming Chang, Shih-Ming Jung, Ming-Huang Chen, Chun-Nan Yeh, Michael Hsiao

**Affiliations:** 1 Department of Medical Research, Tungs’ Taichung Metro Harbor Hospital, Taichung, Taiwan; 2 Graduate Institute of Clinical Medicine, College of Medicine, Taipei Medical University, Taipei, 11031 Taiwan; 3 Genomics Research Center, Academia Sinica, 128 Academia Road, Section 2, Nankang, Taipei 11529 Taiwan; 4 Pulmonary Research Center, Wan Fang Hospital, Taipei Medical University, Taipei, Taiwan; 5 Traditional Herbal Medicine Research Center, Taipei Medical University Hospital, Taipei, Taiwan; 6 TMU Research Center of Cancer Translational Medicine, Taipei Medical University, Taipei, Taiwan; 7 Division of Pulmonary Medicine, Department of Internal Medicine, Wan Fang Hospital, Taipei Medical University, Taipei, Taiwan; 8 School of Oral Hygiene, College of Oral Medicine, Taipei Medical University, Taipei, Taiwan; 9 Department of Pathology, Chang Gung Memorial Hospital, Chang Gung University, Taoyuan, Taiwan; 10 Department of Oncology, Taipei Veterans General Hospital and School of Medicine, National Yang-Ming University, Taipei, 112 Taiwan; 11 School of Medicine, National Yang-Ming University, Taipei, Taiwan; 12 Department of General Surgery and Liver Research Center, Linkou Branch, Chang Gung Memorial Hospital, Chang Gung University, Taoyuan, 333 Taiwan; 13 Department of Biochemistry, Kaohsiung Medical University, Kaohsiung, Taiwan; 14 Graduate Institute of Cancer Biology and Drug Discovery, College of Medical Science and Technology, Taipei Medical University, Taipei, Taiwan

**Keywords:** Hepatocellular carcinoma, Mass spectrometric imaging, Transmembrane P24 trafficking protein 9, Vascular invasion, Prognosis

## Abstract

**Background:**

Due to the difficulties in early diagnosing and treating hepatocellular carcinoma (HCC), prognoses for patients remained poor in the past decade. In this study, we established a screening model to discover novel prognostic biomarkers in HCC patients.

**Methods:**

Candidate biomarkers were screened by liquid chromatography with tandem mass spectrometry (LC-MS/MS) analyses of five HCC normal (N)/tumor (T) paired tissues and preliminarily verified them through several in silico database analyses. Expression levels and functional roles of candidate biomarkers were respectively evaluated by immunohistochemical staining in N/T paired tissue (*n* = 120) and MTS, colony formation, and transwell migration/invasion assays in HCC cell lines. Associations of clinicopathological features and prognoses with candidate biomarkers in HCC patients were analyzed from GEO and TCGA datasets and our recruited cohort.

**Results:**

We found that the transmembrane P24 trafficking protein 9 (TMED9) protein was elevated in HCC tissues according to a global proteomic analysis. Higher messenger (m)RNA and protein levels of TMED9 were observed in HCC tissues compared to normal liver tissues or pre-neoplastic lesions. The TMED9 mRNA expression level was significantly associated with an advanced stage and a poor prognosis of overall survival (OS, *p* = 0.00084) in HCC patients. Moreover, the TMED9 protein expression level was positively correlated with vascular invasion (*p* = 0.026), OS (*p* = 0.044), and disease-free survival (*p* = 0.015) in our recruited Taiwanese cohort. In vitro, manipulation of TMED9 expression in HCC cells significantly affected cell migratory, invasive, proliferative, and colony-forming abilities.

**Conclusions:**

Ours is the first work to identify an oncogenic role of TMED9 in HCC cells and may provide insights into the application of TMED9 as a novel predictor of clinical outcomes and a potential therapeutic target in patients with HCC.

**Supplementary Information:**

The online version contains supplementary material available at 10.1186/s12929-021-00727-5.

## Background

Hepatocellular carcinoma (HCC) is the most fatal malignancy disease among primary liver cancers and is one of the leading causes of cancer-related deaths worldwide [1]. HCC develops from underlying chronic liver disease and cirrhosis, which are usually caused by hepatitis B and C viral (HBV and HCV) infections, alcohol consumption, or diabetes and nonalcoholic fatty liver disease [2–4]. The incidence of HCC is highest in Asia, including Taiwan, and Africa, and it occurs in males more often than in females [5]. The median survival time of HCC in Taiwan is significantly higher than that in Africa, where the median survival is only 2.5 months [6]. To the present, several therapeutic strategies have been applied as HCC treatment, including chemoradiotherapy, surgery, and systemic therapy with sorafenib [7, 8], but low therapeutic and diagnostic efficiencies still lead to severe mortality of patients with this disease. Thus, we wanted to illuminate novel biomarkers for HCC diagnoses or therapeutic targets.

Images from computed tomography (CT) or magnetic resonance imaging (MRI) are key approaches for evaluating the progression of HCC and formulating future treatment plans [9]. In addition, a high serum alpha-fetoprotein (AFP) level has also been widely used for HCC diagnoses and recurrence predictions. However, due to its low sensitivity and specificity, serum AFP is not a highly accurate biomarker for HCC diagnoses [10]. Recently, systemic proteomic analyses have shown promise in exploring proper biomarkers for predicting cancer prognoses [11]. For example, matrix-assisted laser desorption/ionization (MALDI)-imaging mass spectrometry (IMS), multiple reaction monitoring (MRM) MS and liquid chromatography-tandem MS (LC-MS/MS) have been used to discover diagnostic, prognostic and survival biomarkers in liver cancer [12–15]. Although MS protein expression data in HCC are available from recently published works, the prognostic values and functional roles of these proteins in clinical HCC samples and HCC cells have not yet been investigated [12, 14, 16].

Herein, we mined candidate biomarkers of HCC from five paired normal and HCC tissues using LC-MS/MS analyses. Among the candidate proteins, overexpression of transmembrane P24 trafficking protein 9 (TMED9) was observed in HCC samples and pre-neoplastic lesions compared to normal tissues. HCC patients with TMED9^high^ tumors had a higher frequency of developing an advanced stage and vascular invasion, and had shorter overall survival (OS) and disease-free survival (DFS) times compared to patients with TMED9^low^ tumors. Moreover, TMED9-knockdown significantly inhibited the growth and motility of HCC cell lines. Taken together, this study first identified that TMED9 might be a novel prognostic biomarker and therapeutic target for HCC.

## Material and methods

### Cell culture

The human Mahlavu and HCC36 cell lines were respectively maintained in the Dulbecco's modified Eagle and minimum essential medium (DMEM and MEM; Gibco, Waltham, MA, USA) with 10% fetal bovine serum (FBS), 2 mM glutamine, 100 units/mL penicillin, and 100 mg/mL streptomycin, and was incubated in a 5% CO_2_ humidified atmosphere at 37 °C. The TMED9 short hairpin (sh)RNA constructs were purchased from the National RNAi core Facility at Academic Sinica (Taipei, Taiwan). The target sequences were GCC AGT CTT CTG TCT TCC TTT and CGG CAC CTC AAG AGC TTC TTT. The HA-TMED9 construct was purchased form Applied Biological Materials Inc.

### In-gel digestion

Coomassie dye staining gels were cut into small pieces and washed with distilled water (d_2_H_2_O). Gel pieces were destained with 25 mM ammonium bicarbonate (ABC) overnight at 4 °C, which was then replaced with reduction buffer (10 mM dithiothreitol (DTT)/25 mM ABC) for 1 h at 56 °C. Next, the reduction buffer was discarded, and alkylation buffer (55 mM iodoacetamide/25 mM ABC) was added for 1 h at room temperature in the dark and washed twice with 40% acetonitrile (ACN) for 10 min and then dehydrated by treatment with 100% ACN. Dry pieces were re-swollen with 0.12 μg of modified trypsin in 25 mM ABC and digested overnight at 37 °C. Peptides were harvested using 60% ACN/0.1% trifluoroacetic acid (TFA) and sonication. After the solution was dried in a vacuum, the peptides were re-dissolved in 0.1% TFA for the LC-MS/MS analysis.

### LC-MS/MS analysis

LC-MS/MS analyses were performed on an LTQ-FT ion trap mass spectrometer (LTQ FT Ultra, Thermo Scientific, Waltham, MA, USA). The Mascot software package (Matrix Science, Boston, MA, USA) was used for protein identification according to SWISS-PROT, an annotated protein sequence database [17]. The peptide mass tolerance and fragment mass tolerance were set at 100 ppm and 0.25 Da, respectively. Matches with scores higher than the 95% confidence level were regarded as significant.

### Cell-proliferation and colony-formation assays

Mahlavu and HCC36 cells were stably infected with virus carrying either shTMED9, HA-TMED9, or their respective controls. For the proliferation assay, Mahlavu (5 × 10^3^) and HCC36 (6 × 10^3^) cells were seeded into 96-well dishes. After 48 (for HCC36) or 72 h (for Mahlavu), Alamar blue dye was added to each well and incubated at 37 °C for 3 h. The absorbance was measured at 570 nm using a microplate reader (MQX200; Bio-Tek Instruments, Winooski, VT, USA). Values are the mean ± standard deviation (SD) of triplicate wells and were normalized to that of the control group to determine the multiples of proliferative ability.

For the colony-formation assay, 1.5 × 10^3^ Mahlavu cells were seeded into 6-well dishes. After 7–10 days, cells were fixed with methanol and then stained with 1% crystal violet. Numbers of colonies were further quantified by Image J software (National Institutes of Health, Bethesda, MD, USA).

### Transwell migration and Matrigel-invasion assays

For the cell-migration analysis, 10^5^ HCC36 cells infected with virus carrying the Luc-control or HA-TMED9 were plated in an uncoated top chamber (24-well insert; pore size, 8 μm; Corning Costar, Corning, NY). For the cell-invasion analysis, 1.5 × 10^4^ Mahlavu cells infected with virus carrying the sh-control or shTMED9 were seeded in a 20 μg Matrigel (BD Biosciences, Bedford, MA)-coated top chamber. In both assays, top chamber all contained serum-free medium and medium supplemented with serum was used as a chemoattractant in the lower chamber. After 48 and 24 h of incubation, migrated and invaded cells in the lower chamber were fixed with methanol and stained with 0.1% crystal violet. The number of cells migrating through or invading through the membrane was counted under a light microscope (× 100, five random fields per well).

### Bioinformatics analysis

We collected the GSE6764, GSE76311, and GSE102079 microarray datasets, which contain transcriptome profiles of normal tissues, dysplastic tissues, and HCC tissues, from the Gene Expression Omnibus (GEO) database [18–20]. Representative figures for the RNA sequencing analysis were obtained from the visualization platform Gene Expression Profiling Interactive Analysis (GEPIA) (http://gepia.cancer-pku.cn/) [21]. Further, the website STRING (https://string-db.org/) was utilized to explore protein–protein interaction networks of TMED9-regulated genes, and the cBioPortal (https://www.cbioportal.org/) was used to investigate correlations of TMED9 with other genes. Correlations of TMED3 with TMED9 and with patient prognosis data were calculated and downloaded from UCSC Xena (https://xena.ucsc.edu/) [22].

### Patients and tissue microarray (TMA) construction

This study is a hospital-based case–control study. A total of 182 patients who had undergone surgical resection of HCC at Taipei Veterans General Hospital in Taipei, Taiwan were recruited as a case group between 1990 and 2006, with an average follow-up time of 60.02 months. The patients were diagnosed with HCC according to the characteristic criteria of the national guidelines for HCC. Collection of patient data was approved by the Ethics Committee of Taipei Veterans General Hospital (201010021IC). We constructed a formalin-fixed, paraffin-embedded TMA composed of 182 HCC tissue cores. The clinicopathologic data of 182 patients with HCC were obtained by a retrospective review of the medical records. Before 2002, patients with tumor recurrence could receive transarterial chemoembolization, surgery, and a percutaneous ethanol injection. After 2002, other therapeutic choices, such as radiofrequency tumor ablation and liver transplantation, were provided for recurrent patients. Because approval of targeted therapy or immunotherapy was not available in 1990 to 2006, no patients in this study received that drug. Collection of our Taiwanese HCC cohort was described in detail in our previous study [23].

### Immunohistochemical (IHC) staining

IHC staining was automatically performed with an immunostainer (Ventana Discovery XT autostainer, Tucson, AZ, USA). TMA slides were stained with a polyclonal rabbit anti-human TMED9 antibody (GeneTex, Hsinchu, Taiwan), and cytoplasmic expression in HCC cells was evaluated. The staining intensity and percentage of TMED9 were recorded for further analysis. According to the intensity of staining, samples were scored from 0 (no staining) to 3 (heavy staining). The percentage of positive cells was calculated to range 0–100%. The intensity score was multiplied by the percentage of positive cells as the final IHC score (0–300). The high or low expression of TMED9 in HCC patients was divided by a cutoff value of 60.

### Western blotting

Protein extraction and Western blot preparation were described in a previous study [24]. The primary antibodies used in this study were TMED9 (1:1000; GeneTex, Hsinchu, Taiwan); TGF-α (1:250; Santa Cruz, CA, USA), p-ERK (1:1000; Santa Cruz, CA, USA), GLI1 (1:1000; Santa Cruz, CA, USA), β-catenin (1:1000; Cell Signaling, MA, USA), p-STAT3 (1:1000; Cell Signaling, MA, USA), t-STAT3 (1:1000; Cell Signaling, MA, USA), t-ERK (1:1000; Cell Signaling, MA, USA), and GAPDH (1:5000, Sigma, St. Louis, MO, USA).

### Dot blot analysis

Briefly, 5 × 10^5^ HCC cells were seeded in a 6-cm Petridish and infected with virus carrying either shTMED9, HA-TMED9, or their respective controls. After infection, the cell culture medium was replaced by serum-free medium for another 24 h and harvested for dot blotting. Each dot was presented the proteins in 300 µL soup. The hybridization and detection were followed by Western blot protocol.

### Statistical analysis

All spectra were processed by baseline subtraction, peak detection, and peak-area calculation according to default settings. Discrimination between control and tumor samples was performed using an algorithm. Values from in vitro studies are presented as the mean ± standard deviation (SD). Data were analyzed using Student’s *t*-test when two groups were compared. Correlations of TMED9 with clinicopathologic features of HCC were examined by Pearson’s Chi-squared test. Cumulative survival was analyzed by the Kaplan–Meier method. Risk factors affecting survival were assessed by a Cox proportional hazards regression model. A *p* value of < 0.05 was considered a statistically significant difference.

## Results

### Potential biomarkers of HCC are discovered by the LC–MS/MS analysis

**Fig. 1:**
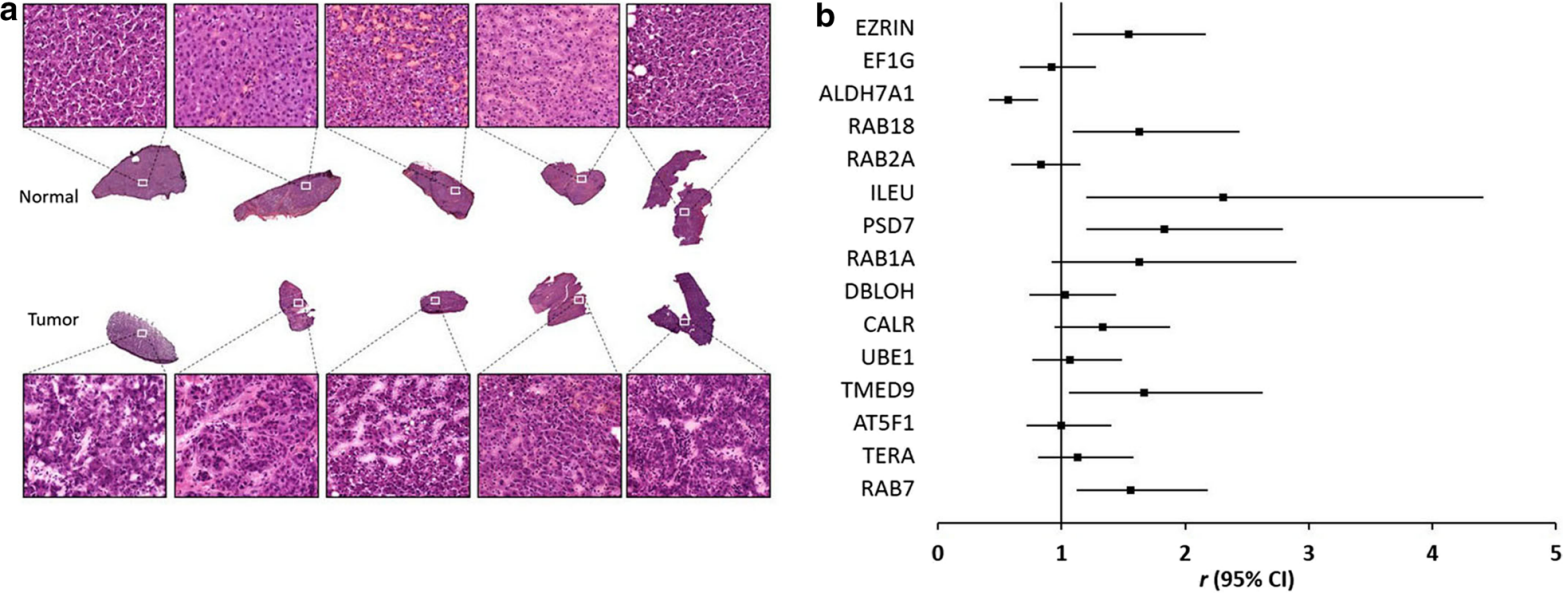
Clinical values of candidate genes derived from an LC–MS/MS analysis of paired normal (N)/tumorous (T) hepatocellular carcinoma (HCC) samples. **a** Representative H&E staining pictures of the histomorphology of normal and HCC samples. **b** Forest plot showing hazard ratios (HRs) and 95% confidence intervals for the association of candidate genes and overall survival in patients with HCC

In this study, we first applied sodium dodecyl sulfate polyacrylamide gel electrophoresis (SDS-PAGE) coupled with LC-MS/MS to identify candidate biomarkers from five sets of paired HCC N/T tissues. Fresh protein lysates from paired N/T tissues were separated by SDS-PAGE and analyzed by LC/MS/MS. Then, protein IDs were identified by a Mascot engine through searching the SWISS-PROT database, and protein expression levels were calculated according to Mascot scores. Hematoxylin and eosin (H&E) staining of samples was used by a pathologist to define the cancer and non-cancerous parts (Fig. 1a). By interpretation of the protein ID analysis and a literature search, we selected several candidate proteins which had a > 1 and those with a < 1 score ratio of HCC tumor versus normal samples (Table 1, Additional file 1: Table S1).

**Table 1:** List of protein score ratios (> 1) of hepatocellular carcinoma (HCC) tumor (T) versus normal (N) samples

Protein ID	Mass	T/N	SE
Ezrin	69,370	4.73	0.99
Elongation factor 1-gamma	50,087	4.01	0.81
Aldehyde dehydrogenase family 7 member A1	55,331	3.51	0.90
Calcium-binding mitochondrial carrier protein Aralar2	74,129	2.84	0.48
Ras-related protein Rab-18	22,963	2.68	0.39
Ras-related protein Rab-2A	23,531	2.41	0.23
Leukocyte elastase inhibitor (LEI) (Serpin B1)	42,715	2.24	0.30
26S proteasome non-ATPase regulatory subunit 7	37,002	2.23	0.15
Ras-related protein Rab-1A	22,663	1.83	0.26
Proteasome subunit beta type 5 precursor	22,882	1.81	0.19
Diablo homolog, mitochondrial precursor	27,114	1.75	0.11
4-trimethylaminobutyraldehyde dehydrogenase	53,767	1.69	0.15
Calreticulin precursor	48,112	1.59	0.11
Ubiquitin-activating enzyme E1	117,774	1.55	0.27
Transmembrane emp24 domain-containing protein 9 precursor	25,089	1.47	0.12
Cytochrome c	11,741	1.45	0.15
ATP synthase subunit b	28,890	1.43	0.06
Transitional endoplasmic reticulum ATPase	89,266	1.20	0.09
Ras-related protein Rab-7	23,475	1.19	0.14
UMP-CMP kinase	22,208	1.38	0.19

*SE* standard error

We next validated the prognostic values of these 20 HCC tissue-upregulated candidates by online databases, including SurvExpress (http://bioinformatica.mty.itesm.mx/SurvExpress) and TCGA (https://tcga-data.nci.nih.gov) [22, 25]. Most of these 20 candidate proteins exhibited higher mRNA levels in HCC tissues compared to normal liver tissues according to the GEPIA analysis (http://gepia.cancer-pku.cn/index.html) (Table 2). According to the OS analysis by the SurvExpress website, we further found that elevated mRNA expression of some candidates correlated with a poor prognosis in HCC patients, especially Ezrin, Rab18, ILEU (SERPINB1), PSD7 (PSMD7), TMED9, and Rab7 (Fig. 1b). Among these, Rab1a and Rab18 had previously been reported to promote HCC cell proliferation or act as a poor prognostic marker in HCC patients [26, 27]. Ezrin overexpression was associated with vascular invasion and a poor prognosis in patients with HCC [28, 29]. These results indicated the high credibility and applicability of our screening system to search for candidate biomarkers in HCC patients.

**Table 2:** mRNA expression levels of candidate proteins in hepatocellular carcinoma (HCC) tissues

Protein ID/*Gene name*	Gepia T (TPM)	Gepia N (TPM)
Ezrin/*EZR*	21.5	18.64
Elongation factor 1-gamma/*EF1G*	721.42	356.44
Aldehyde dehydrogenase family 7 member A1/*ALDH7A1*	112.76	74.83
Calcium-binding mitochondrial carrier protein Aralar2/*SLC25A13*	33.92	34.11
Ras-related protein Rab-18/*RAB18*	21.87	14.14
Ras-related protein Rab-2A/*RAB2A*	62.71	36
Leukocyte elastase inhibitor (LEI)/*SERPINB1*	26.38	10.95
26S proteasome non-ATPase regulatory subunit 7/*PSMD7*	42.62	28.45
Ras-related protein Rab-1A/*RAB1A*	84.1	65.7
Proteasome subunit beta type 5 precursor/*PSMB5*	103.42	47.67
Diablo homolog, mitochondrial precursor/*DIABLO*	40.03	23.54
4-trimethylaminobutyraldehyde dehydrogenase/*ALDH9A1*	52.42	48.4
Calreticulin precursor/*CALR*	813.78	337.32
Ubiquitin-activating enzyme E1/*UBA1*	67.5	40.51
Transmembrane emp24 domain-containing protein 9 precursor/*TMED9*	183.3	91.03
Cytochrome c/*CYC*	157.32	75.33
ATP synthase subunit b/*ATP5F1*	161.42	101.54
Transitional endoplasmic reticulum ATPase/*VCP*	101.63	60.53
Ras-related protein Rab-7/*Rab7A*	87.32	58.97
UMP-CMP kinase/*CMPK1*	56.7	54.61

*T* tumor tissue, *N* normal tissue, *TPM* transcripts per million

### mRNA expression level of TMED9 is significantly higher in HCC tumor tissues and is correlated with an advanced stage and poor patient prognoses

**Fig. 2:**
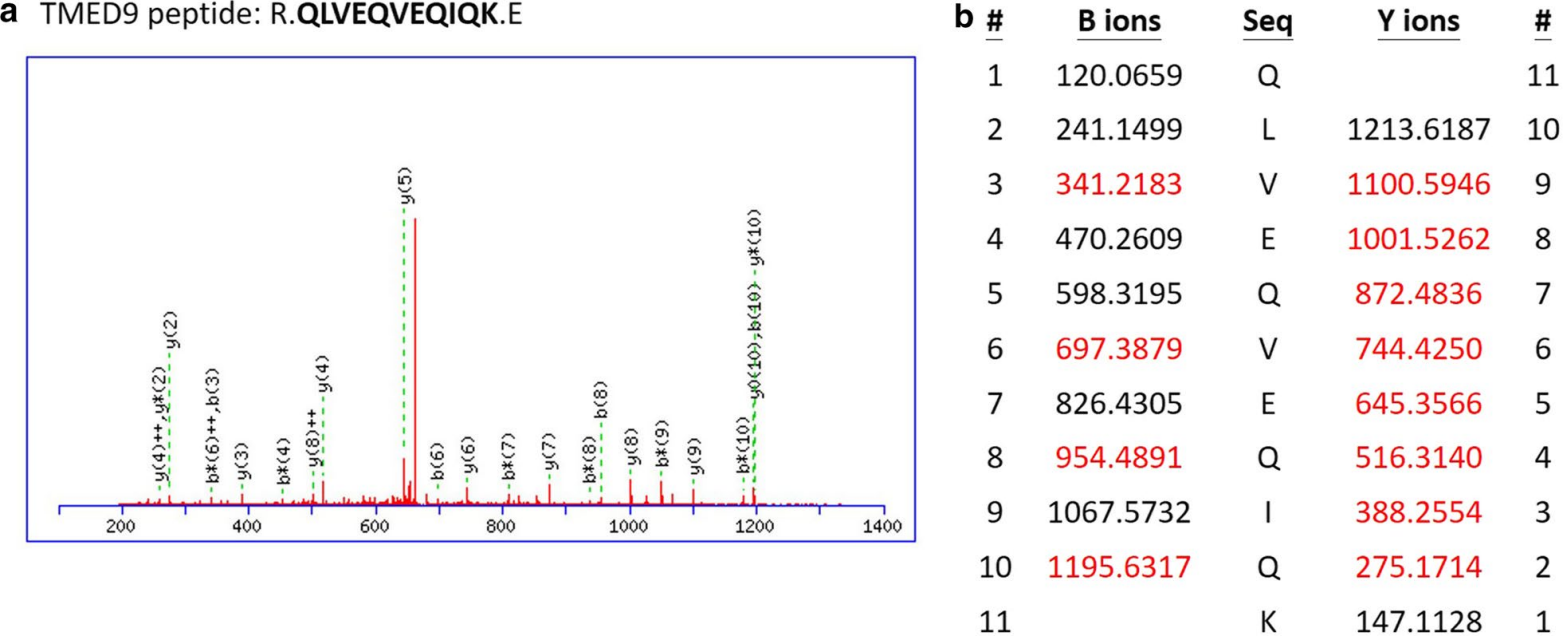
Results of an LC–MS/MS analysis for TMED9. **a** Representative data showing the sequence of the trypsinized peptide, R.QLVEQVEQIQK.E, which was matched to TMED9 in hepatocellular carcinoma (HCC) patient samples. **b** Matching B-ions and Y-ions (red) fragments were recorded to identify the sequence of TMED9. The retained charge on the amino-terminal part of the peptide is shown as B-ions, and the retained charge on the carboxyl-terminal part of the peptide is Y-ions

**Fig. 3:**
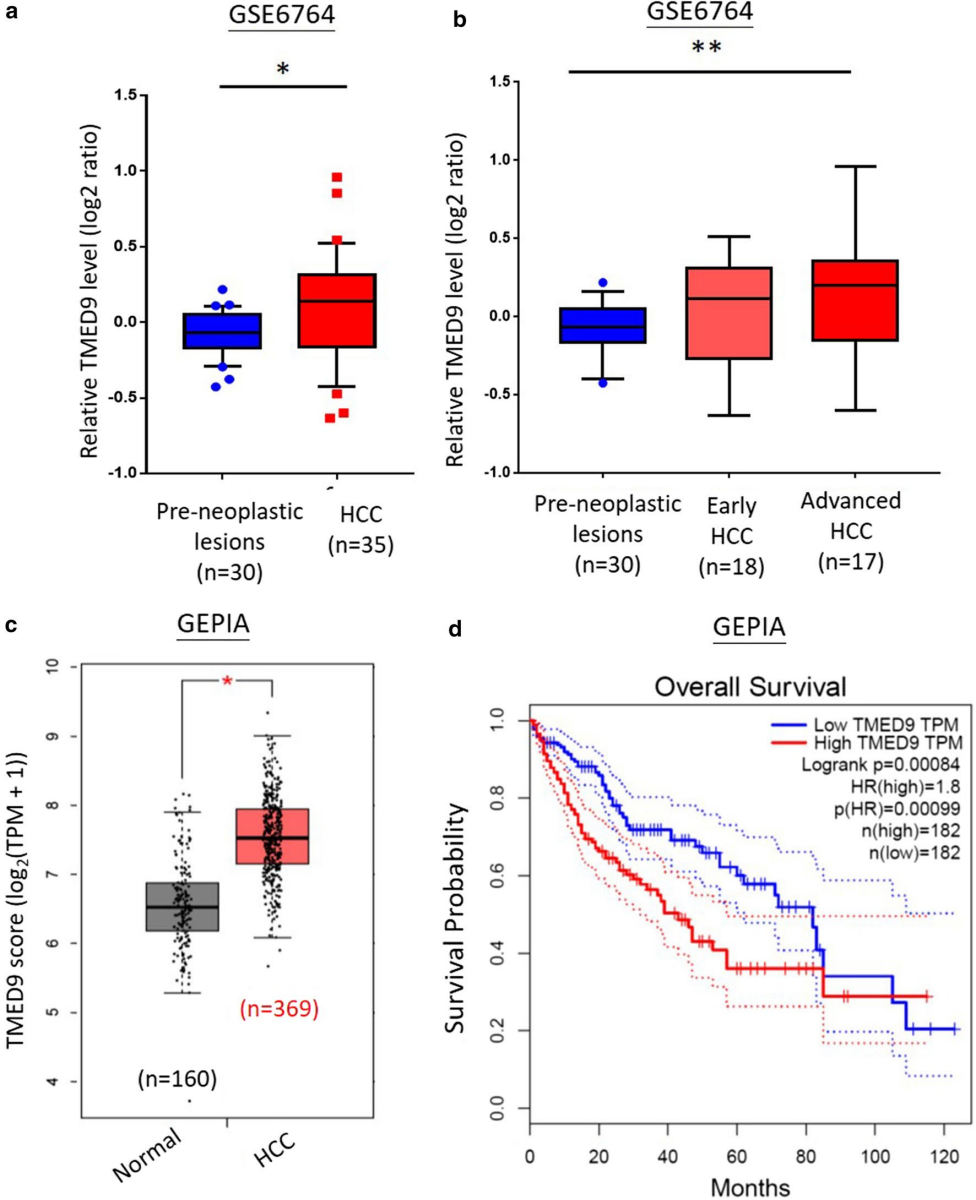
In silico analysis of TMED9 expression in hepatocellular carcinoma (HCC). **a**, **b** Relative TMED9 mRNA levels (208757_at) in 30 pre-neoplastic lesions (including 13 cirrhotic samples and 17 dysplastic samples) and 35 HCC (including 18 early-stage and 17 advanced-stage HCC) samples from GSE6764 microarray datasets. Expression data were normalized to the median intensity of all probes and scaled to log2 transformation. **c** Expression levels of TMED9 transcripts in normal and HCC samples. **d** Kaplan–Meier curves for overall survival of patients with HCC, as categorized according to high or low expression of TMED9. The *p* value indicates a comparison between patients with TMED9^high^ and TMED9^low^. Data in both **c** and **d** are from GEPIA online available databases (http://gepia.cancer-pku.cn/index.html)

Among these 20 candidate proteins, the role of TMED9, a protein secretion modulator, in HCC is largely unexplored. According to LC–MS/MS data, several peptides belonging to TMED9 were identified in patient samples (Additional file 1: Table S2). For instance, the LC–MS/MS fragment of R.QLVEQVEQIQK.E was identified as TMED9_HUMAN (Fig. 2). Thus, we hypothesized that TMED9 could be a putative prognostic biomarker in HCC. To further identify the potential prognostic value of TMED9 in HCC, we first analyzed its expression levels in 30 pre-neoplastic lesions (including 13 cirrhotic and 17 dysplastic samples) and 35 HCC samples (including 18 early and 17 advanced HCC) from the GSE6764 dataset of the GEO database. Significantly higher TMED9 transcripts were observed in tumors samples, especially in advanced HCC, than in pre-neoplastic lesions (Fig. 3a, b). In addition, we also examined TMED9 levels in paired N/T tissues in the GSE76311 dataset (Additional file 2: Figure S1a) and normal tissue versus tumor tissue in GSE102079 dataset (Additional file 2: Figure S1b). These results indicated significantly elevated TMED9 transcripts in malignant tissues. In addition to the GEO database, we then explored TMED9 in HCC via the GEPIA website (http://gepia.cancer-pku.cn/detail.php) based on the TCGA and GTEx databases. We also observed that TMED9 transcripts were more highly expressed in HCC tumor samples compared to normal liver tissues (Fig. 3c). Moreover, the prognostic value of TMED9 was also verified by GEPIA, and results showed that the OS of HCC patients was adversely affected by a higher TMED9 expression level (*p* = 0.00084) (Fig. 3d).

### Expression of TMED9 protein is significantly elevated in HCC tissues, and high TMED9 expression is correlated with vascular invasion and poor prognoses in HCC patients

**Fig. 4:**
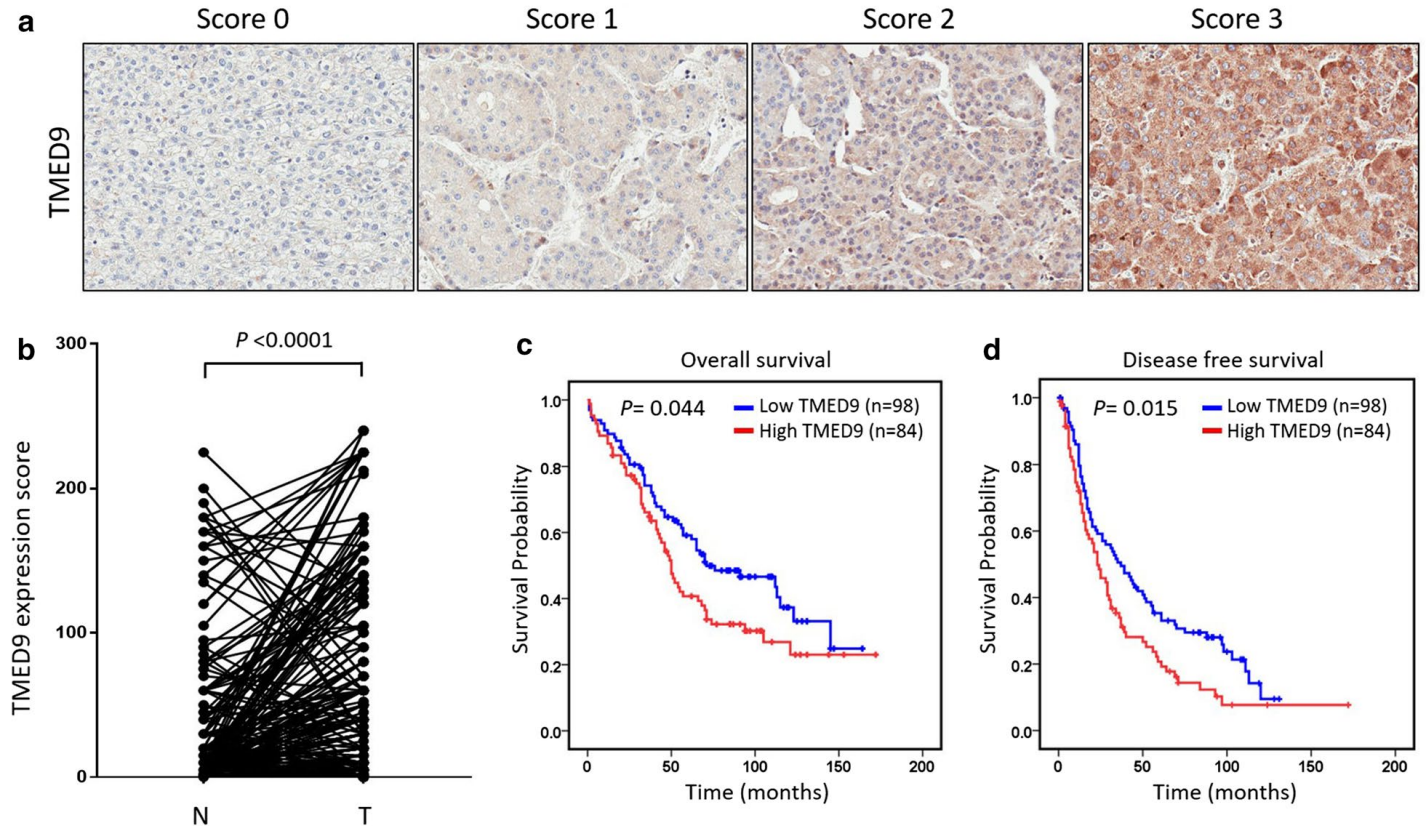
TMED9 expression correlates with overall and disease-free survival in patients with hepatocellular carcinoma (HCC) after surgical resection. **a** Tissue microarray of HCC was immunohistochemically analyzed for TMED9 and showed different intensity scores for TMED9 expression. **b** TMED9 expression scores were significantly higher in tumor than non-tumor tissues. Statistical significance was analyzed by a paired *t* test (*p* < 0.0001). **c**, **d** Kaplan–Meier plot of overall **c** and disease-free **d** survival of 182 patients with HCC stratified by TMED9 expression levels. A log-rank test was used to show differences between groups

In addition to analyzing mRNA levels of TMED9 from datasets available online, we further verified protein levels of TMED9 by IHC staining from a Taiwanese HCC tissue array cohort. Representative examples of tumors showing overall negative (score 0), low (score 1), moderate (score 2), and high TMED9 (score 3) intensities are illustrated in Fig. 4a, and all positive cases revealed a diffuse cytoplasmic TMED9 distribution in cancer cells. According to our analysis, the score of TMED9 in HCC was significantly higher than that in corresponding non-cancerous tissues (*p* < 0.0001) (Fig. 4b). We thus stratified patients into high- and low-TMED9 groups, and high expression levels of TMED9 were significantly correlated with worse OS (*p* = 0.044; Fig. 4c) and DFS (*p* = 0.015; Fig. 4d) in our recruited cohort.

Clinicopathological parameters of HCC patients are shown in Table 3. There were 182 patients, including 155 males and 27 females. Survival follow-up was available in all cases and ranged 1 ~ 172 months (median, 52 months; mean, 60.02 months). Among these patients, 43 cases (23.6%) experienced no recurrence, and 139 cases (76.4%) had disease recurrence during the follow-up period. A Chi-squared analysis was conducted to explore relationships between selected clinicopathologic features and TMED9 expression. Table 3 shows that the TMED9 expression level was significantly correlated with hepatic vein invasion (*p* = 0.026), but not other clinicopathologic features such as the AFP level, HBV, liver cirrhosis, tumor size, and so on.

**Table 3:** Relationship between TMED9 expression and clinicopathological features in 182 patients with hepatocellular carcinoma (HCC)

Characteristic	TMED9 expression		*p* value
	Low (*n* = 98)	High (*n* = 84)	
Age, years (mean ± SD)	61.7 ± 11.0	60.85 ± 13.65	
Gender			
Male	83	72	0.847
Female	15	12	
AFP (ng/mL)			
< 400	79	63	0.293
≥ 400	17	20	
Unknown	2	1	
Stage			
I + II	72	57	0.406
I II	26	27	
HBV			
Negative	35	26	0.407
Positive	60	58	
Unknown	3	0	
HCV			
Negative	63	67	0.024
Positive	33	16	
Unknown	2	1	
Recurrence status			
No	26	17	0.319
Yes	72	67	
Liver cirrhosis			
No	51	45	0.872
Yes	41	38	
Unknown	6	1	
Tumor size (cm)			
< 5	57	47	0.764
> 5	41	37	
Tumor numbers			
Solitary	57	48	0.89
Multiple	41	36	
Hepatic vein invasion			
No	95	74	0.026
Yes	2	8	
Unknown	1	2	
Portal vein invasion			
No	89	72	0.186
Yes	7	11	
Unknown	2	1	

*SD* standard deviation, *AFP* alpha fetoprotein, *HBV* hepatitis B virus, *HCV* hepatitis C virus

Moreover, we utilized a univariate analysis to examine the prognostic significance of clinicopathologic variables in our HCC cohort. In addition to TMED9 expression (hazard ratio (HR), 1.463; *p* = 0.047), the tumor size (HR, 1.543; *p* = 0.023), portal vein invasion (HR, 3.463; *p* < 0.001), and hepatic vein invasion (HR, 3.971; *p* < 0.001) were all shown to have adverse impacts on OS (Table 4). Furthermore, DFS was also adversely affected by high TMED9 expression (HR, 1.506; *p* = 0.017), portal vein invasion (HR, 3.662; *p* < 0.001), and hepatic vein invasion (HR, 11.333; *p* < 0.001) (Table 5).

**Table 4:** Cox univariate and multivariate regression analysis of prognostic factors and TMED9 expression for overall survival (OS) in 182 hepatocellular carcinoma (HCC) patients

Variables	Comparison	HR (95% CI)	*P* value
Cox Univariate Analysis (OS)			
Age	< 61 years; ≥ 61 years	1.233 (0.836–1.818)	0.292
Gender	Female; male	0.695 (0.397–1.218)	0.204
Stage	I; II; III	1.255 (0.951–1.657)	0.109
AFP	< 400 ng/mL; ≥ 400 ng/mL	1.398 (0.887–2.203)	0.149
HBV	Negative; positive	1.111 (0.763–1.617)	0.584
HCV	Negative; positive	0.917 (0.603–1.396)	0.687
Tumor size	≤ 5 cm; > 5 cm	1.543 (1.061–2.244)	0.023*
Cut margin	< 1 cm; > 1 cm	0.728 (0.488–1.086)	0.120
PVI	No; yes	3.463 (1.993–6.014)	0.000***
HVI	No; yes	3.971 (1.980–7.964)	0.000***
TMED9	Low; high	1.463 (1.005–2.128)	0.047*
Cox Multivariate Analysis (OS)			
Tumor size	≤ 5 cm; > 5 cm	1.323 (0.883–1.981)	0.175
PVI	No; yes	2.444 (1.216–4.911)	0.012*
HVI	No; yes	1.580 (0.656–3.809)	0.308
TMED9	Low; high	1.249 (0.831–1.876)	0.285

*HR* hazard ratio, *CI* confidence interval, *AFP* alpha fetoprotein, *HBV* hepatitis B virus, *HCV* hepatitis C virus, *PVI* portal venin invasion, *HVI* hepatic vein invasion

*p < 0.05, **p < 0.01,  ***p < 0.001

**Table 5:** Cox univariate and multivariate regression analysis of prognostic factors and TMED9 expression for disease-free survival (DFS) in 182 hepatocellular carcinoma (HCC) patients

Variables	Comparison	HR (95% CI)	*P* value
Cox Univariate Analysis (DFS)			
Age	< 61 years; ≥ 61 years	0.814 (0.583–1.138)	0.229
Gender	Female; male	0.866 (0.549–1.366)	0.536
Stage	I; II; III	1.248 (0.977–1.594)	0.076
AFP	< 400 ng/mL; ≥ 400 ng/mL	1.159 (0.753–1.782)	0.503
HBV	Negative; positive	1.262 (0.897–1.777)	0.182
HCV	Negative; positive	1.090 (0.753–1.577)	0.649
Tumor size	≤ 5 cm; > 5 cm	1.371 (0.979–1.920)	0.066
Cut margin	< 1 cm; > 1 cm	0.686 (0.481–0.979)	0.038*
PVI	No; yes	3.662 (2.043–6.567)	0.000***
HVI	No; yes	11.333 (5.191–24.138)	0.000***
TMED9	Low; high	1.506 (1.076–2.107)	0.017*
Cox Multivariate Analysis (DFS)			
Cut margin	≤ 1 cm; > 1 cm	0.770 (0.525–1.129)	0.181
PVI	No; yes	2.438 (1.268–4.688)	0.008**
HVI	No; yes	8.273 (3.531–19.381)	< 0.001***
TMED9	Low; high	1.173 (0.807–1.703)	0.403

All terms are defined in the legend to Table 4

### TMED9 modulates the cell-proliferative, colony-forming, and invasive/migratory abilities of HCC cells

**Fig. 5:**
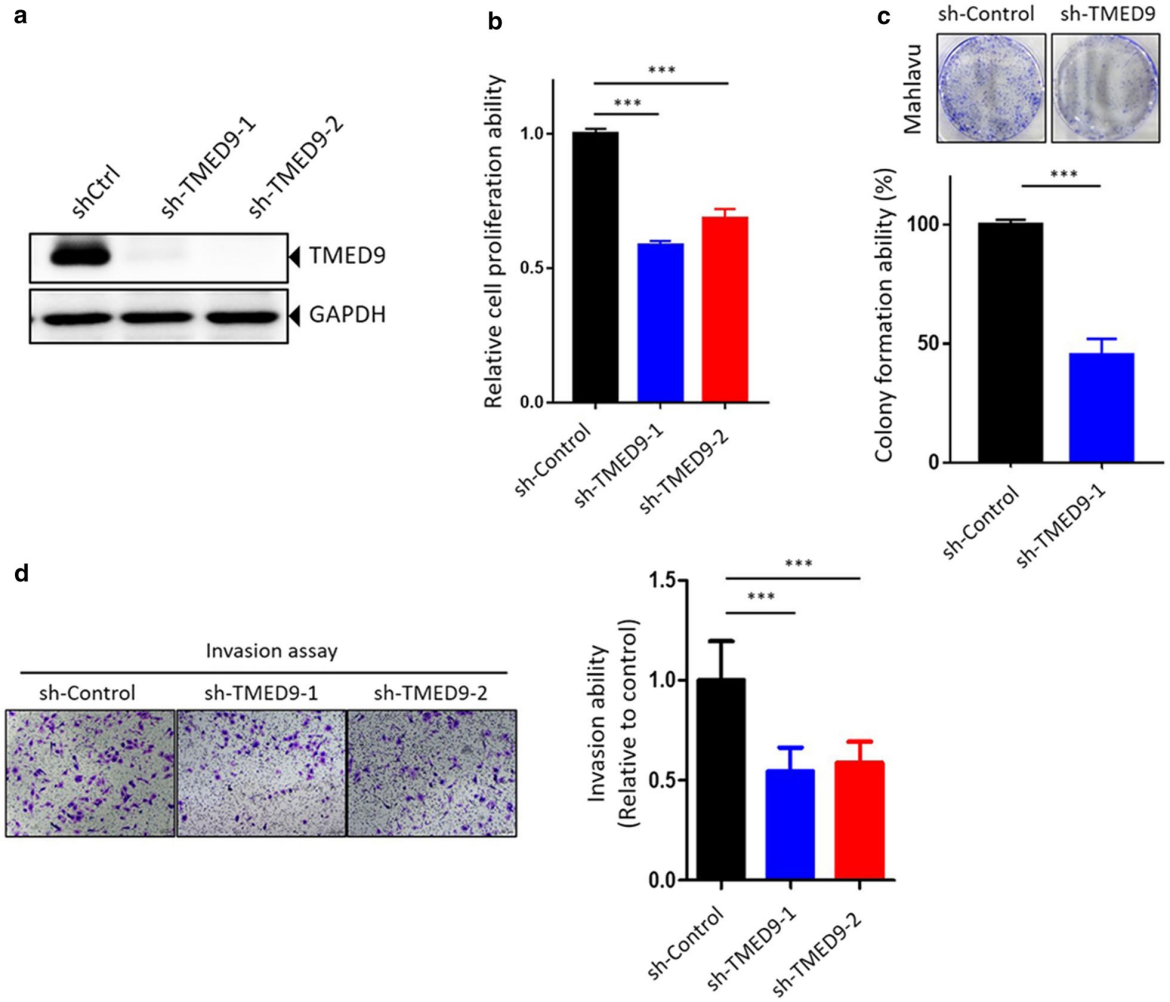
Depletion of TMED9 decreases cell growth and invasion abilities of hepatocellular carcinoma (HCC) cells. **a** Mahlavu cells were infected with a lentivirus carrying control shRNA (sh-Ctrl) or two TMED9 shRNAs (shTMED9). After 72 h, knockdown efficiencies of the two TMED9 shRNAs were determined by Western blotting. **b**–**d** Cell-proliferative, colony-forming, and invasive abilities of TMED9-knockdown Mahlavu cells were respectively determined by Alamar blue cell-viability (**b**), colony-formation (**c**), and transwell-invasion **d** assays. Upper panel of **C** and left panel of **D**: representative photomicrographs. Values are presented as the mean ± SD from three independent experiments. ***p < 0.001 compared to the sh-Ctrl group

To further investigate the functional role of TMED9 in HCC cells, we knocked-down TMED9 by two lentiviral-based shRNAs in highly invasive Mahlavu HCC cells (Fig. 5a) and examined the cell-invasive, proliferative, and colony-forming abilities of HCC cells. As shown in Fig. 5b, a significantly lower proliferative ability of HCC cells was observed in TMED9-knockdown groups than in control groups. Moreover, we examined the effect of TMED9 on the long-term growth (10 days) of Mahlavu cells using a colony-formation assay and found that the number of colonies was reduced following TMED9 knockdown (Fig. 5c). Furthermore, results from the transwell invasion assay showed that significant attenuation of the invasive ability was observed in TMED9-depleted HCC cells (Fig. 5d). In comparison to Mahlavu cells, we ectopically expressed TMED9 in low-invasive HCC36 cells (Additional file 2: Figure S2a) and observed that overexpression of TMED9 significantly increased cell proliferation (Additional file 2: Figure S2b) and motility (Additional file 2: Figure S2c). These results indicated that TMED9 may be deeply involved in HCC progression, and suggested that targeting TMED9 might be a potential therapeutic strategy for arresting HCC progression.

## Discussion

The main finding of the present study was to identify TMED9, one of the transport proteins located in the endoplasmic reticulum (ER) and Golgi network, as a prognostic biomarker of HCC progression. Reliable mass spectrometry-based analyses have been used for drug discovery, metabolomic analysis, and cancer biomarker investigations [30, 31]. Herein, we interpreted LC–MS/MS data to identify elevated candidate proteins in HCC tissues (Table 1) for diagnostic and prognostic predictions in HCC. Similar to TMED9, one of the candidate markers, aldehyde dehydrogenase 7 family member A1 (ALDH7A1), was previously reported to be more highly expressed in HCC tissues than in non-tumorous liver tissues, but its expression in tumor tissues was not correlated with OS, recurrence, or microvascular invasion [32]. Although some candidate markers, such as Rab1, Rab18, and Ezrin which are elevated in HCC, were reported to promote HCC progression [26–29], the oncogenic or tumor-suppressive roles of other candidate proteins we found in HCC tissues remain unclear. For example, Rab7 expression regulated the migratory ability of lung cancer H1299 cells through Ras-related C3 botulinum toxin substrate 1 (RAC1) and vimentin [33]. In MCF-7 breast cancer cells, Rab7 is responsible for Akt survival signal maintenance to promote cells anoikis resistance [34]. In contrast, Rab7 was reported to be a tumor suppressor in prostate cancer based on the fact that it inhibited ligand-induced c-Met signaling [35]. In addition to Rab7, it was revealed that overexpression of serine protease inhibitor B1 (SERPINB1; also known as ILEU) promotes the motility of oral squamous cell carcinoma [36], but the epigenetic suppression of SERPINB1 predicts a poorer prognosis in prostate cancer patients [37]. Thus, further investigations of the roles of candidate proteins which we found in HCC tissues are still needed. After a literature review and our preliminary in silico analysis, we found that TMED9 showed a poor prognostic impact on HCC, but has rarely been investigated in HCC.

TMED9 belongs to the transmembrane emp24 domain-containing protein (TMED)/p24 family, including TMED1–7 and TMED9–11 which are responsible for selecting cargo in the processing ER-Golgi network of proteins (secretory pathway) and innate immune signaling [38, 39]. Given that a huge diversity exists among the proteins of this family, TMED proteins can influence the stability of each other while forming monomers or dynamic complexes [40, 41]. Abnormalities in the controlled transport of proteins in the secretory pathway contribute to diseases such as cancer. For example, a reduction in TMED2 was reported to increase the possibility of liver tumorigenesis [39]. In contrast, elevated levels of TMED2 were observed in patients with ovarian and breast cancers. TMED2 was identified as a promoter of cell proliferation and migration in ovarian cancer cells through activating Akt and as a poor prognostic factor in patients with breast cancer [42, 43]. TMED3 was reported to be an oncoprotein in HCC, prostate cancers, breast cancers, and kidney cancers. [44–47]. For example, TMED3 enhanced cell migration by activating interleukin (IL)-11/signal transducer and activator of transcription 3 (STAT3) signaling and promoted HCC progression [47]. TMED3 was also reported to promote proliferation and motility of breast cancer cells by activating WNT/β-catenin pathway [48]. In contrast, TMED3 was identified as a tumor suppressor in colon cancer and proposed to inhibit metastasis by repressing TMED9. TMED3 depletion can trigger a TMED9-induced metastasis via inducing transforming growth factor (TGF)-α secretion and upregulating CNIH4/TGF-α/GLI1 signaling [49]. According to those results, the malignant properties of TMED2 and TMED3 in cancer are cell type-specific.

**Fig. 6:**
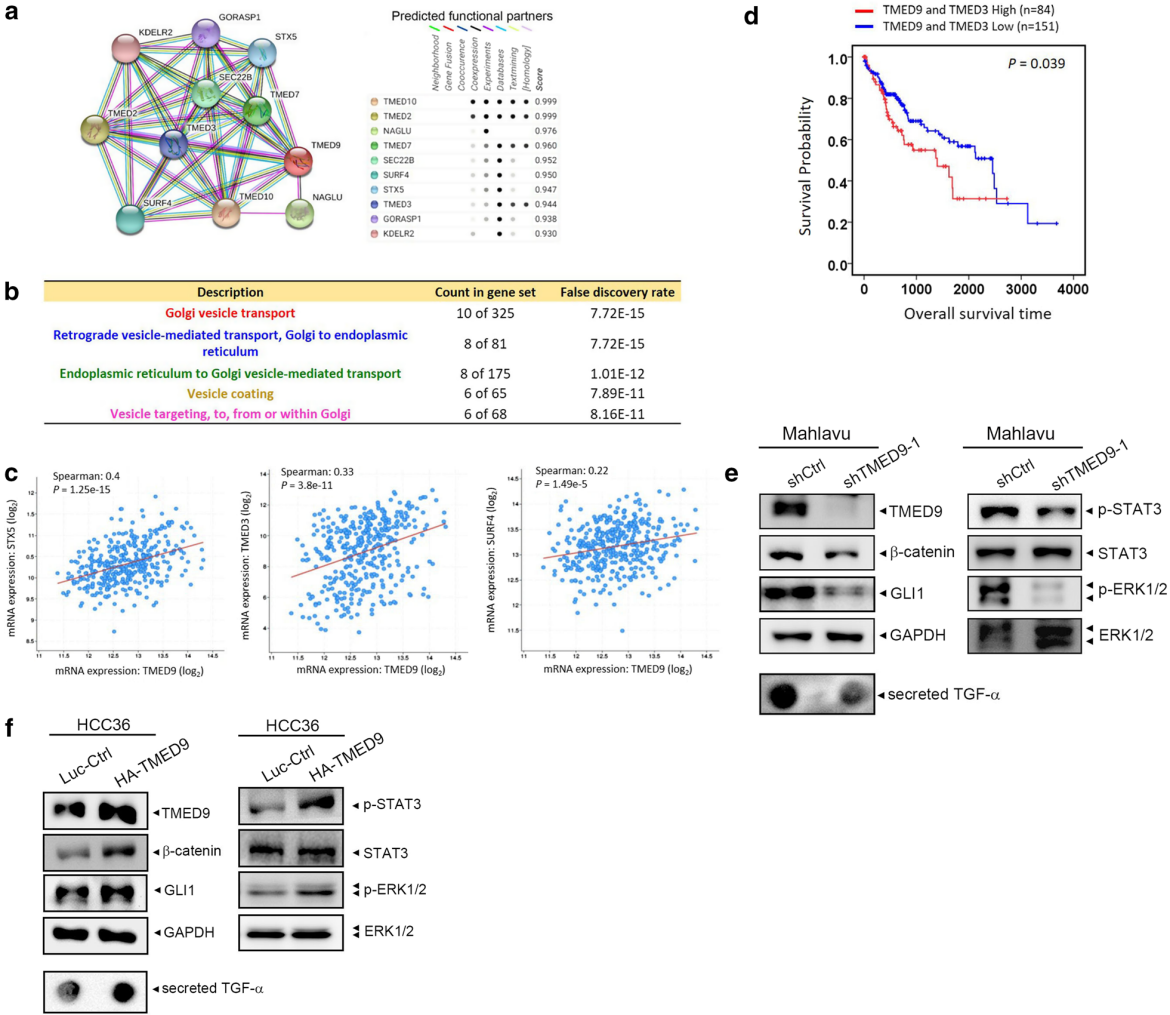
Prediction of potential interactions between cargo-transport proteins with TMED9 and their prognostic values in hepatocellular carcinoma (HCC). **a** TMED9 protein–protein interaction network of 10 differentially expressed genes from the STRING database. **b** Gene ontology analysis revealed that TMED9 participates in endoplasmic reticular (ER)-Golgi cargo trafficking. **c** Correlation analysis of The Cancer Genome Atlas (TCGA) Liver Hepatocellular Carcinoma database using the cBioPortal revealed positive correlations between TMED9 and mRNA levels of STX5, TMED3, and SURF4. **d** Effects of TMED9 and TMED3 mRNA levels on patient overall survival from the TCGA cohort. **e**, **f** Mahlavu (**e**) and HCC36 (**f**) cells respectively expressed shTMED9 and HA-TMED9 as indicated. Cell lysates and conditioned media were harvested to determine the expression of β-catenin and GLI1, phosphorylation of ERK and STAT3, and secretion of TGF-α in both cells by Western blotting and dot blot analyses

TMED2 and TMED3 were respectively reported to be a tumor suppressor and an oncogene in HCC [39, 47]. However, the oncogenic role of TMED9 was only identified in colon cancer [49], but not in other cancer types. In this study, we first reported the prognostic and functional roles of TMED9 in HCC. We further dissected interacting neighbors of TMED9 using the STRING database (https://string-db.org/) and found ten potential TMED9-associated genes, including TMED10, TMED2, TMED3, TMED7, STX5, KDELR2, SEC22B, SURF4, GORASP1, and NAGLU (Fig. 6a), which are necessary for Golgi vesicle transport and related vesicle-trafficking functions (Fig. 6b). We then verified correlations of TMED9 with these genes in 373 HCC human samples using the cBioportal platform and observed that TMED9 expression was only significantly correlated with STX5, TMED3, and SURF4 (Fig. 6c). Furthermore, TCGA database showed that a poorer prognosis was observed in HCC patients with high levels of TMED9 and TMED3 in tumors compared to patients with low levels of TMED9 and TMED3 in tumors (Fig. 6d). Opposite roles of TMED9 and TMED3 in regulating the progression of colon cancer were reported [49]. Although the roles of TMED9 and TMED3 in HCC differed from those in colon cancer, how the interaction between TMED9 and TMED3 promotes HCC progression remains for further evaluation. Herein, we first evaluated the effect of TMED9 knockdown on TMED9- and TMED3-modulated signaling molecules which have been reported in other cancer types [48, 49]. We observed that TMED9 knockdown in Mahlavu cells decreased expression of β-catenin and GLI1, phosphorylation of ERK and STAT3, and secretion of TGF-α compared to control cells (Fig. 6e). In comparison, β-catenin, GLI1, p-ERK, p-STAT3, and secreted TGF-α were all upregulated in TMED9-overexpressed HCC36 cells (Fig. 6f). These results suggested that CNIH4/TGF-α/GLI1 and WNT/β-catenin pathways might be involved in TMED9-induced HCC progression and these issues should be further investigated in future research.

Our current study is not without limitations. First, since the HCC samples were collected from 1990 to 2006 in our study, the examination of HBV/HCV viral levels was not been determined and no anti-viral therapies were available at that period. Thus, we can't analyze whether the baseline viral level affecting HCC patient outcome. Moreover, the sample size of the current study was still not large enough and might lead to a limited statistical impact on the accuracy and precision of the results. For example, TMED9 expression level has a negative impact on OS and DFS from univariate, but not multivariate analysis. Larger HCC cohort should be further recruited to confirm this result in future. Furthermore, the clinical data and follow-up conditions were prospectively collected. Even we did our best to collect detail clinical information, there were still few patients lacking the status of cirrhosis or hepatic/portal vein invasion.

## Conclusions

According to our study, TMED9 is present in fresh-frozen HCC tissues and can be successfully detected by an LC-MS/MS analysis. Using this technology, we first identified that TMED9 could be a valuable prognostic biomarker for HCC and revealed the functional role of TMED9 in HCC cells. Whether TMED9 can interact with TMED3 or other cargo-transport proteins to promote HCC cell proliferation and motility needs be further addressed; yet our present findings strongly support the targeting of TMED9 as a novel strategy for HCC treatment.

## Supplementary Information

**Additional file 1: Table S1.** List of protein score ratio (< 1) of hepatocellular carcinoma (HCC) tumor versus normal samples. **Table S2.** Matched TMED9 peptides in hepatocellular carcinoma (HCC) tissues.

**Additional file 2: Figure S1.** Expression of TMED9 transcripts in paired adjacent (GSE76311, probe: TC05001018.hg.1; **a** and unpaired normal and tumor tissues (GSE102079, probe: 208757_at; **b** derived from patients with hepatocellular carcinoma (HCC). **Figure S2.** TMED9 overexpression promotes cell growth and migration of HCC36 cells. **a** HCC36 cells were infected with a lentivirus carrying control vector or HA-TMED9. After 72 h, the expression of TMED9 was determined by Western blotting. **b**, **c** Cell-proliferative **b** and migratory **c** abilities of HCC36 cells expressing control vector or HA-TMED9. Values are presented as the mean ± SD from three independent experiments. *p < 0.05; **p < 0.01 versus the vehicle control group.

## Data Availability

All data used during the current study are available from the corresponding author on reasonable request.

## Abbreviations

ABC: Ammonium bicarbonate
ACN: Acetonitrile
DFS: Disease-free survival
DTT: Dithiothreitol
GEO: Gene Expression Omnibus
GEPIA: Gene Expression Profiling Interactive Analysis
GTEx: Genotype-Tissue Expression
HCC: Hepatocellular carcinoma
HR: Hazard ratio
IHC: Immunohistochemical/immunohistochemistry
LC–MS/MS: Liquid chromatography-tandem mass spectrometry
OCT: Optimum cutting temperature
OS: Overall survival
TCGA: The Cancer Genome Atlas
TMED9: Transmembrane P24 trafficking protein 9
TPM: Transcripts per million
