# Supplementary material for: Proteomics-based identification of TMED9 is linked to vascular invasion and poor prognoses in patients with hepatocellular carcinoma
Source: J Biomed Sci. 2021 Apr 22;28:29. doi: 10.1186/s12929-021-00727-5 (PMC8063382; doi:10.1186/s12929-021-00727-5)
Supplement: Supplementary file 1 — Additional file 1: Table S1. List of protein score ratio (< 1) of hepatocellular carcinoma (HCC) tumor versus normal samples. Table S2. Matched TMED9 peptides in hepatocellular carcinoma (HCC) tissues. [file 12929_2021_727_MOESM1_ESM.docx]

**Additional Information**

**Title:**

**Proteomics-based identification of TMED9 is linked to vascular invasion and poor prognoses in patients with hepatocellular carcinoma**

Yi-Chieh Yang, Ming-Hsien Chien*, Tsung-Ching Lai, Min-Che Tung, Yi-Hua Jan, Wei-Ming Chang, Tsung-Hui Hu, Ming-Huang Chen*, Chun-Nan Yeh*, Michael Hsiao*

^*^Correspondence to: Dr. Ming-Hsien Chien (E-mail: mhchien1976@gmail.com), Ming-Huang Chen (E-mail: mhchen9@gmail.com), Dr. Chun-Nan Yeh (E-mail: ycn@cgmh.org.tw) and Dr. Michael Hsiao (E-mail: mhsiao@gate.sinica.edu.tw)

**Additional file 1: Table S1.** List of protein score ratios (<1) of hepatocellular carcinoma (HCC) tumor (T) versus normal (N) samples

| Protein ID | Mass | T/N | SE |
| --- | --- | --- | --- |
| Formimidoyltransferase-cyclodeaminas | 58889 | 0.34 | 0.04 |
| Fructose-bisphosphate aldolase B | 39448 | 0.35 | 0.03 |
| Annexin A4 | 35860 | 0.39 | 0.08 |
| Glutathione S-transferase P | 23341 | 0.42 | 0.06 |
| Carbonyl reductase [NADPH] 1 | 30356 | 0.44 | 0.03 |
| Cofilin-1 | 18491 | 0.45 | 0.09 |
| Glyceraldehyde-3-phosphate dehydrogenase | 36030 | 0.48 | 0.05 |
| Annexin A5 | 35914 | 0.57 | 0.10 |
| Fibrinogen beta chain precursor | 55892 | 0.58 | 0.06 |
| Ferritin light chain | 20007 | 0.62 | 0.08 |
| 4-Aminobutyrate aminotransferase, mitochondrial precursor | 56403 | 0.64 | 0.07 |
| NADP-dependent leukotriene B4 12-hydroxydehydrogenase | 35847 | 0.64 | 0.12 |
| Ribosome-binding protein 1 | 152381 | 0.65 | 0.10 |
| 2,4-Dienoyl-CoA reductase, mitochondrial precursor | 36045 | 0.67 | 0.11 |
| UDP-glucose 6-dehydrogenase | 54989 | 0.71 | 0.17 |
| Mitochondrial carrier homolog 2 | 33309 | 0.72 | 0.07 |
| Peptidyl-prolyl cis-trans isomerase B precursor | 22728 | 0.73 | 0.15 |
| Heat shock cognate 71 kDa protein | 70854 | 0.81 | 0.21 |

**Additional file 1: Table S2.** Matched TMED9 peptides in hepatocellular carcinoma (HCC) tissues

| **Query** | **Mr (calc)** | **Score** | **Peptide** |
| --- | --- | --- | --- |
| 84 | 727.35 | 33 | K.SFFEAK.K |
| 944 | 1097.57 | 54 | K.FSLFAGGMLR.V |
| 957 | 1100.62 | 39 | K.DKLSELQLR.V |
| 1943 | 1340.73 | 76 | R.QLVEQVEQIQK.E |
