# Supplementary material for: Proteomics-based identification of TMED9 is linked to vascular invasion and poor prognoses in patients with hepatocellular carcinoma
Source: J Biomed Sci. 2021 Apr 22;28:29. doi: 10.1186/s12929-021-00727-5 (PMC8063382; doi:10.1186/s12929-021-00727-5)
Supplement: Supplementary file 2 — Additional file 2: Figure S1. Expression of TMED9 transcripts in paired adjacent (GSE76311, probe: TC05001018.hg.1; a and unpaired normal and tumor tissues (GSE102079, probe: 208757_at; b derived from patients with hepatocellular carcinoma (HCC). Figure S2. TMED9 overexpression promotes cell growth and migration of HCC36 cells. a HCC36 cells were infected with a lentivirus carrying control vector or HA-TMED9. After 72 h, the expression of TMED9 was determined by Western blotting. b, c Cell-proliferative b and migratory c abilities of HCC36 cells expressing control vector or HA-TMED9. Values are presented as the mean ± SD from three independent experiments. *p < 0.05; **p < 0.01 versus the vehicle control group. [file 12929_2021_727_MOESM2_ESM.docx]

**
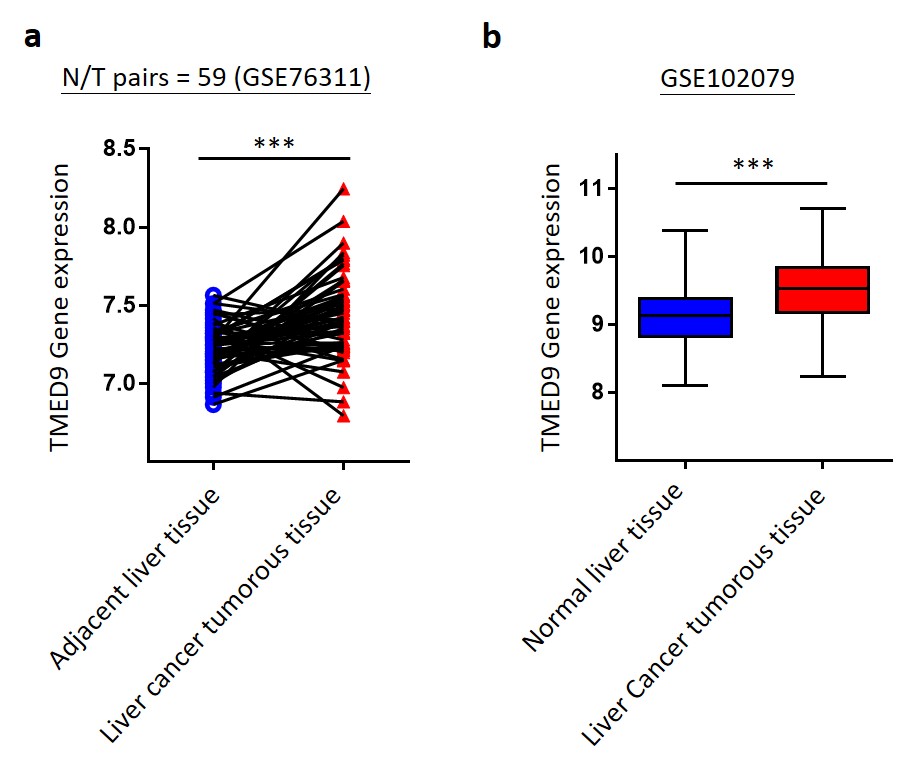
**

**Additional file 2: Figure S1.** Expression of TMED9 transcripts in paired adjacent (GSE76311, probe: TC05001018.hg.1; a) and unpaired (GSE102079, probe: 208757_at; b) normal and tumor tissues derived from patients with hepatocellular carcinoma (HCC).


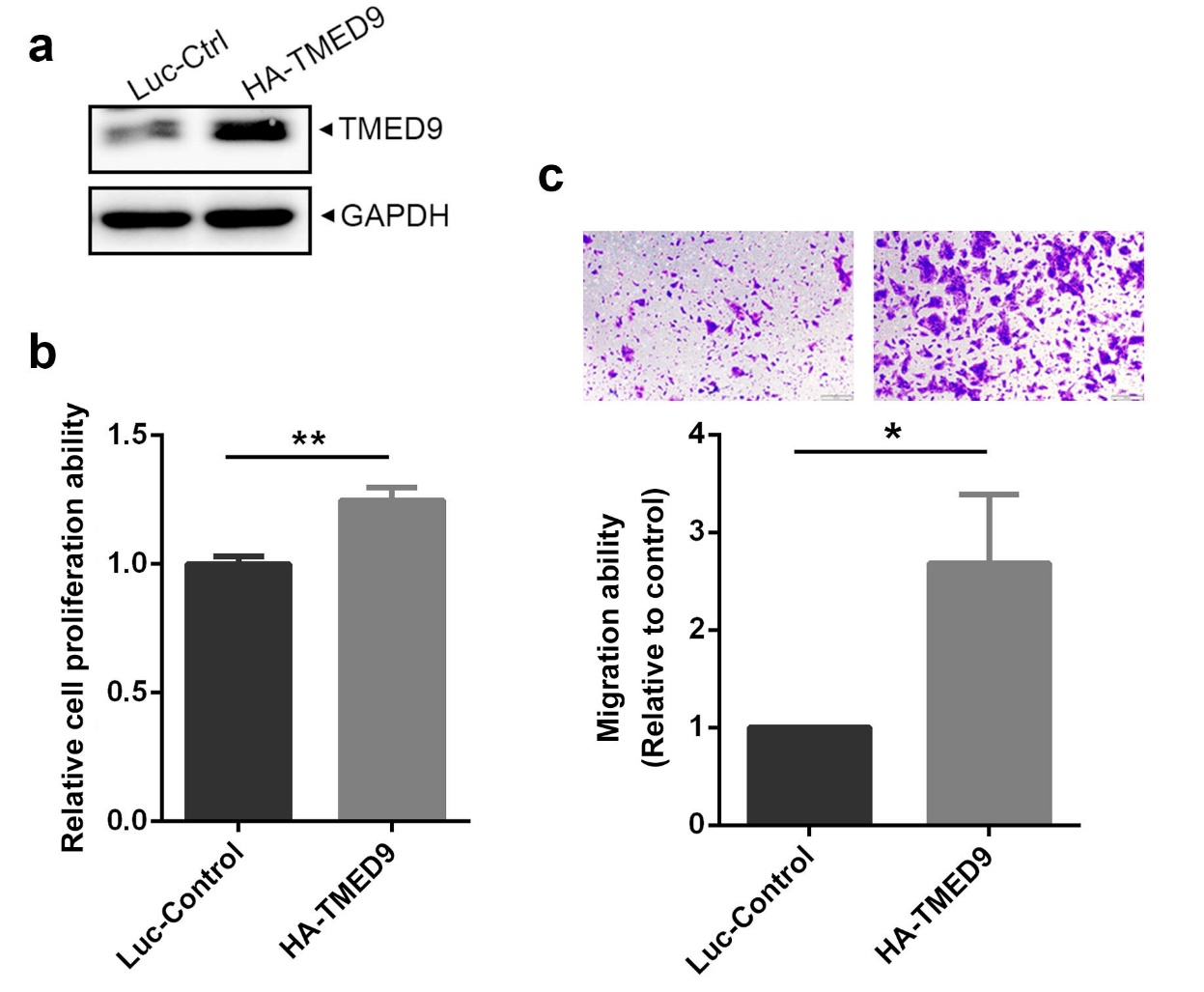


**Additional file 2: Figure S2. TMED9 overexpression promotes cell growth and migration of HCC36 cells.** (a) HCC36 cells were infected with a lentivirus carrying control vector or HA-TMED9. After 72 h, the expression of TMED9 was determined by Western blotting. (b-c) Cell-proliferative (b) and migratory (c) abilities of HCC36 cells expressing control vector or HA-TMED9. Values are presented as the mean ± SD from three independent experiments. * *p* < 0.05; ** *p* < 0.01 versus the vehicle control group.
